# Clinical efficacy of Yiqi Yangxue formula on knee osteoarthritis and unraveling therapeutic mechanism through plasma metabolites in rats

**DOI:** 10.3389/fgene.2023.1096616

**Published:** 2023-04-05

**Authors:** Ting Zhao, Shiqi Wang, Wenbin Liu, Jiayan Shen, Youwu Dai, Mingqin Shi, Xiaoyi Huang, Yuanyuan Wei, Tao Li, Xiaoyu Zhang, Zhaohu Xie, Na Wang, Dongdong Qin, Zhaofu Li

**Affiliations:** ^1^ The First School of Clinical Medicine, Yunnan University of Chinese Medicine, Kunming, China; ^2^ The Central Hospital of Enshi Tujia and Miao Autonomous Prefecture, Enshi, China; ^3^ School of Basic Medical Sciences, Yunnan University of Chinese Medicine, Kunming, China; ^4^ Qujing Hospital Affiliated to Yunnan University of Traditional Chinese Medicine, Qujing, China; ^5^ Institutes of Integrative Medicine, Fudan University, Shanghai, China

**Keywords:** knee osteoarthritis, YQYXF, clinical efficacy, metabolome, biomarkers

## Abstract

**Objective:** To observe the clinical efficacy and safety of Yiqi Yangxue formula (YQYXF) on knee osteoarthritis (KOA), and to explore the underlying therapeutic mechanism of YQYXF through endogenous differential metabolites and their related metabolic pathways.

**Methods:** A total of 61 KOA patients were recruited and divided into the treatment group (YQYXF, 30 cases) and the control group (celecoxib, Cxb, 31 cases). Effects of these two drugs on joint pain, swelling, erythrocyte sedimentation rate (ESR) and c-reactive protein (CRP) were observed, and their safety and adverse reactions were investigated. In animal experiments, 63 SD rats were randomly divided into normal control (NC) group, sham operation (sham) group, model (KOA) group, Cxb group, as well as low-dose (YL), medium-dose (YM), and high-dose groups of YQYXF (YH). The KOA rat model was established using a modified Hulth method. Ultra-high-performance liquid chromatography/Q Exactive HF-X Hybrid Quadrupole-Orbitrap Mass (UHPLC-QE-MS)-based metabolomics technology was used to analyze the changes of metabolites in plasma samples of rats. Comprehensive (VIP) >1 and *t*-test *p* < 0.05 conditions were used to screen the disease biomarkers of KOA, and the underlying mechanisms of YQYXF were explored through metabolic pathway enrichment analysis. The related markers of YQYXF were further verified by ELISA (enzyme-linked immunosorbent assay).

**Results:** YQYXF can improve joint pain, swelling, range of motion, joint function, Michel Lequesen index of severity for osteoarthritis (ISOA) score, Western Ontario and McMaster Universities Osteoarthritis Index (WOMAC) score, ESR, and CRP. No apparent adverse reactions were reported. In addition, YQYXF can improve cartilage damage in KOA rats, reverse the abnormal changes of 16 different metabolites, and exert an anti-KOA effect mainly through five metabolic pathways. The levels of reactive oxygen species (ROS) and glutathione (GSH) were significantly decreased after the treatment of YQYXF.

**Conclusion:** YQYXF can significantly improve the clinical symptoms of KOA patients without obvious adverse reactions. It mainly improved KOA through modulating lipid metabolism-related biomarkers, reducing lipid peroxidation and oxidative stress.

## Introduction

Knee osteoarthritis (KOA) is the most prevalent form of arthritis characterized by a degeneration of articular cartilage resulting in the development of osteophytes, or bone spurs (Horecka et al., 2022). The global prevalence of KOA is around 3.8% (Kao et al., 2022). KOA is closely associated with age, as radiographic evidence of KOA occurs in most people over the age of 65 years (Lespasio et al., 2017). Furthermore, the incidence of KOA increases with a higher average weight of the population, particularly in obese women (Zhang and Jordan, 2010). Strenuous physical activity, especially activities requiring kneeling, knee-bending, squatting, and prolonged standing, as well as knee trauma and injury have also been linked to a high prevalence of KOA (Heidari, 2011).

Although the mechanisms of degenerative changes are better-understood thanks to numerous biochemical and genetic studies, drugs that can stop the degenerative cascade remain unknown. So far, arthritis have been managed pharmacologically and non-pharmacologically, including common pharmacotherapies, surgery, and lifestyle changes. All available forms of KOA therapy are based on symptomatic treatment, such as pain relief and joint function improvement (Nowaczyk et al., 2022). Pain medications, including the most popular non-steroidal anti-inflammatory drugs (NSAIDs), are the first-line treatment (Gnylorybov et al., 2020). Surgery should be considered only in the case of no improvement and the presence of advanced lesions visible in imaging tests. Currently, an increasing number of studies are being published suggesting that traditional Chinese medicine may be as effective or even more effective than NSAIDs and result in fewer systemic adverse effects (Glyn-Jones et al., 2015; Wang et al., 2020). Yiqi Yangxue formula (YQYXF) is a prescribed Chinese herbal formula for treating KOA based on the traditional Chinese medicine theory. The YQYXF consists of astragalus, codonopsis, tangerine peel, cohosh, bupleurum, angelica, atractylodes, cassia twig, white peony, licorice, divaricate saposhniovia root, ligusticum wallichii, rhizoma drynariae, epimedium, a total of 14 herbs. In our previous study, YQYXF could inhibit the levels of matrix metalloproteases 1 (MMP-1) and MMP-13, promoting chondrocyte proliferation (Duan R. et al., 2020). However, the potential mechanism of YQYXF in treating KOA is still unclear.

In the present study, we aimed to evaluate the clinical efficacy and safety of YQYXF in patients with KOA. We observed the effects of YQYXF and celecoxib (Cxb) on visual analogue scale (VAS) score, swelling, range of motion (ROM) and joint function, Michel Lequesen index of severity for osteoarthritis (ISOA) score, the Western Ontario and McMaster Universities Osteoarthritis Index (WOMAC), Kellgren-Lawrence score, erythrocyte sedimentation rate (ESR) and c-reactive protein (CRP) index of KOA patients (Kellgren and Lawrence, 1957; Brosseau et al., 2003; Zhang et al., 2009; Anil et al., 2021). The safety and adverse reactions were investigated by blood cell analysis (white blood cell, red blood cell, hemoglobin, and platelet), liver function (alanine transaminase, aspartate aminotransferase), and kidney function (blood urea nitrogen, creatinine) (Wu et al., 2021). In addition, we used the KOA rat model to further analyze the changes of metabolites in rat plasma samples using metabonomics technologies based on ultra-high-performance liquid chromatography/Q Exactive HF-X Hybrid Quadrupole-Orbitrap Mass (UHPLC-QE-MS) (Xiao et al., 2012; Wang et al., 2014). The potential mechanism of YQYXF on endogenous differential metabolites and related metabolic pathways was also discussed, providing evidence for the treatment of KOA.

## Materials and methods

### Clinical study design

#### Sample source and grouping

Sixty-one KOA patients (Kellgren-Lawrence score I-III) were recruited from Yunnan Provincial Hospital of Traditional Chinese Medicine. The patients were divided into a treatment group with 30 patients (YQYXF) and a control group with 31 patients (celecoxib, Cxb). All patients fulfilled the American College of Rheumatology criteria for primary KOA, and the subjects agreed to sign the informed clinical consent. The age was between 38 and 70 years old—patients who discontinued NSAIDs for 7 days or more. The exclusion criteria included patients with other rheumatic diseases, such as rheumatoid arthritis, Sjögren’s syndrome, and gouty arthritis. In addition, patients with allergies or severe other systemic diseases will not participate in this study. The design scheme of this project has been approved by the Medical Ethics Committee of Yunnan University of Traditional Chinese Medicine (ethics number: 2019YXLL005).

#### Experimental drugs and treatments

YQYXF granules, provided by Jiangyin Tianjiang Pharmaceutical Co., Ltd. YQYXF granules consist of astragalus 30 g, codonopsis 15 g, tangerine peel 10 g, cohosh 10 g, bupleurum 10 g, angelica 20 g, cassia twig 15 g, white peony 15 g, atractylodes 15 g, licorice 5 g, divaricate saposhniovia root 15 g, ligusticum wallichii 15 g, rhizoma drynariae 15 g, epimedium 15 g. The Cxb capsules, provided by Ruihui Pharmaceutical Co., Ltd., are approved by Chinese medicine J20080059. In this study, the treatment group was given YQYXF, one bag/time, three times a day. The control group was given 200 mg/day of Cxb capsules once times a day.

#### Observation indicators and methods

All patients were evaluated using the VAS score and ISOA score, WOMAC, Kellgren-Lawrence score, range of motion (ROM), and joint function grades before the treatment and at 4 weeks after treatment (Kellgren and Lawrence, 1957; Brosseau et al., 2003; Zhang et al., 2009; Anil et al., 2021). ESR and CRP were detected before treatment and on the fourth weekend of treatment, respectively. ESR was detected by the ESR analyzer. C-reactive protein (CRP) was detected by immunoturbidimetry. The Kellgren-Lawrence grading system for KOA is the most widely used method and has become a widely accepted method for the diagnosis of KOA, which is a grading method for the severity of KOA. According to X-ray findings of the knee joint, grading of the severity of KOA can be divided into grade 0 (normal knee joint), grade I, grade II, grade III, and grade IV (the most severe KOA) (Kellgren and Lawrence, 1957; Zeng et al., 2017; Di Martino et al., 2022). Grading of joint function can be divided into four grades. Grade I refers to various activities that can be done. Grade II refers to moderate limitation. Although one or more joints are uncomfortable or have limited movement, they can still engage in normal activities. Grade III refers to limited actions and can only take care of themselves but cannot engage in general activities. Grade IV refers to lying in bed or sitting in bed and you cannot take care of yourself (Zheng, 2002). Joint pain (VAS of patient and physician) was scored using a 10-cm visual analog scale (VAS), and the patients were instructed to mark the corresponding position on the VAS that represented their pain (Abbott et al., 2013; Su et al., 2018). 0 cm: no pain; 1–3 cm: mild pain, but still able to engage in normal activities; 4–6 cm: moderate pain, affecting work, but able to take care of themselves; 7–9 cm: severe pain, unable to take care of themselves; 10 cm: extreme pain. The efficacy evaluation, joint function, swelling, and range of motion in this study are determined according to the guiding principles for clinical research of new drugs of traditional Chinese medicine (Zheng, 2002). Highly effective: pain and swelling disappear, joint activity is normal, and the score decreases by ≥ 95%. Moderately effective: pain and swelling disappear, joint activity is not limited, and score decreases ≥70% and <95%. Lowly effective: pain and swelling symptoms are basically eliminated, joint activity is slightly limited, and the score decreased by ≥ 30% and <70%. Ineffective: pain, swelling, and joint range of motion did not improve significantly, and the score decreased by < 30%.

### Animal experimental design

#### Preparation of experimental drugs

The 14 herbs in YQYXF are provided by the Chinese Pharmacy of the First Affiliated Hospital of Yunnan University of Traditional Chinese Medicine, and their composition and dosage are the same as those of YQYXF granules. The high dose group of YQYXF was administered by gavage with an aqueous solution containing 18.4 g of crude drug/kg, the medium dose group was administered with 9.2 g of crude drug/kg, and the low dose group was administered with 4.6 g of crude drug/kg of rats (respectively equivalent to 0.25, 0.5, and 1 time of the human clinical equivalent dose according to the body surface area dose conversion method of humans and rats Meeh-Rubner formula). In the positive drug group, 18 mg Cxb/kg aqueous solution was administered by gavage. Cxb capsules, provided by Ruihui Pharmaceutical Co., Ltd., approved by Chinese medicine J20080059.

#### Identification of the compounds in YQYXF by UHPLC-QE-MS

Compounds in YQYXF were analyzed using a UHPLC system (Vanquish, Thermo Fisher Scientific) equipped with a waters UPLC BEH C18 column (1.7 μm 2.1 100 mm), and the flow rate was set to 0.5 mL/min, and an injection volume was set to 5 μL. Mobile phase A consisted of 0.1% formic acid solution, and mobile phase B was 0.1% formic acid in acetonitrile. The multi-step linear elution gradient program was as follows: 0–11 min, 85%–25% A; 11–12 min, 25%–2% A; 12–14 min, 2%–2% A; 14–14.1 min, 2%–85% A. During each acquisition cycle, the mass range was from 100 to 1,500, the top four of every process were screened, and the corresponding MS/MS data were further acquired. Sheath gas flow rate: 35 Arb, Aux gas flow rate: 15 Arb, Ion Transfer Tube Temp: 350°C, Vaporizer Temp: 350°C, Full ms resolution: 60,000, MS/MS resolution: 15,000, Collision energy: 16/32/48 in NCE mode, Spray Voltage: 5.5 kV (positive) or −4 kV (negative). An Orbitrap Exploris 120 mass spectrometer coupled with Xcalibur software was employed to obtain the MS and MS/MS data information of YQYXF based on the IDA acquisition mode. The raw data of mass spectra were imported into XCMS software for processing, such as retention time correction, peak identification, peak extraction, peak integration, and peak alignment. The peak information of compounds was searched through the in-house secondary mass spectrometry database provided by Shanghai BIOTREE Biotech Co., Ltd.

#### Administration of the KOA rat model

Sixty-three SPF-grade female SD rats (180 ± 20 g) were purchased from Hunan Slike Jingda Laboratory Animal Co., Ltd., license number: SCXK (Xiang) 2019–0004. The experiments were conducted under full authorization from the Ethics Committee of Yunnan University of Chinese Medicine (ethical code no. R-062019065). The rats were randomly divided into seven groups, including normal control (NC), sham-operated (sham), model (KOA), Cxb (18 mg/kg), YQYXF low-, middle-, and high-dose groups (18.4, 9.2, and 4.6 g of crude drug/kg, respectively). Each group had nine rats. The KOA models were established by the modified Hulth method (Rogart et al., 1999). The anterior ligament was severed, and the medial meniscus was removed with the tibial joint reduction (Gu et al., 2021). The knee in the sham group was only treated with joint capsule opening and suturing. The operation was conducted in an aseptic environment. After the successful modeling, the NC group and the sham group were provided with normal drinking water and diet. The rest were given the corresponding drug suspension by gavage once a day for eight consecutive weeks. The plasma was collected by centrifugation, and the right knee joint was taken. The knee joints were fixed with 10% neutral formaldehyde solution and decalcified with 10% EDTA for 56 days. The tissue was embedded in paraffin and sliced with a thickness of 5 μm.

### Hematoxylin-eosin (HE) staining

Routine dewaxing, rinsed with tap water, stained with hematoxylin solution for 5 min, dehydrated in acid water and ammonia, rinsed with tap water, dehydrated and stained with eosin for 3 min. Dehydrated from low to high concentrations of alcohol, clear, sealed with neutral glue.

#### Metabolites extraction and UHPLC-QE-MS analysis

Add 400 μL of extract (methanol: acetonitrile = 1:1 (V/V), containing isotope-labeled internal standard mixture) to 100 μL of plasma and vortex to mix for 30 s. Sonicate for 10 min and let stand at −40°C for 1 h, centrifuge at 12,000 rpm for 15 min at 4°C, and collect the supernatant for assay. The quality control (QC) sample was prepared by mixing an equal aliquot of the supernatants from all plasma samples. UHPLC-QE-MS analyses were performed using a UHPLC system (Vanquish, Thermo Fisher Scientific) with a UPLC BEH Amide column (2.1 mm × 100 mm, 1.7 μm) coupled to Q Exactive HFX mass spectrometer (Orbitrap MS, Thermo). The mobile phase consisted of 25 mmol/L ammonium acetate and 25 mmol/L ammonia hydroxide in water (A) and acetonitrile (B). The auto-sampler temperature was 4°C, and the injection volume was 2 μL. The QE HFX mass spectrometer was used for its ability to acquire MS/MS spectra in information-dependent acquisition mode in the control of the acquisition software (Xcalibur, Thermo). In this mode, the acquisition software continuously evaluated the full scan MS spectrum. The ESI source conditions were set as follows: sheath gas flow rate as 30 Arb, Aux gas flow rate as 25 Arb, capillary temperature as 350°C, full MS resolution as 60,000, MS/MS resolution as 7,500, collision energy as 10/30/60 in NCE mode, spray Voltage as 3.6 kV (positive) or −3.2 kV (negative), respectively. The raw data were converted to the mzXML format using ProteoWizard and processed with an in-house program, which was developed using R and based on XCMS, for peak detection, extraction, alignment, and integration. Then an in-house MS2 database (BiotreeDB) was applied in metabolite annotation. The cutoff for annotation was set at 0.3. The Thermo Q Exactive HFX mass spectrometer is capable of primary and secondary mass spectral data acquisition under the control of the acquisition software (Xcalibur, Thermo).

Principal components analysis (PCA) and orthogonal correction partial least squares discriminant analysis (OPLS-DA) were conducted using the SIMCA16.0.2 software package (Sartorius Stedim Data Analytics AB, Umea, Sweden). PCA, an unsupervised analysis that reduces the dimension of the data, was carried out to visualize the distribution and the grouping of the samples. A 95% confidence interval in the PCA score plot was used as the threshold to identify potential outliers in the dataset. In order to visualize group separation and find significantly changed metabolites, supervised orthogonal projections to latent structures-discriminate analysis (OPLS-DA) were applied. Then, 7-fold cross-validation was performed to examine the quality of the model. Permutation tests were used to test the validity of the model. The first principal component of variable importance in the projection (VIP) and Student’s t-test were obtained to refine the analysis. Suppose VIP>1 and *p* < 0.05, the variable was defined as a significantly different metabolite between the two groups. The significantly different metabolites were used for plotting hierarchical clustering based on the Euclidean distance formula and drawn heat maps using the Pheatmap package in R studio. The volcano plots were used to filter the metabolites of interest based on Log 2 (fold change) and–Log 10 (*p*-value). The Kyoto Encyclopedia of Genes and Genomes (KEGG, http://www.genome.jp/kegg/) and MetaboAnalyst (http://www.metaboanalyst.ca/) databases were used for pathway enrichment analysis.

#### The determination of the content of lipid peroxidation-related indexes

The plasma of the rats was collected, and the levels of lipid peroxidation-related indexes, such as reactive oxygen species (ROS) and glutathione (GSH), were tested by enzyme-linked immunosorbent assay (ELISA). The ELISA kit (Jiangsu Jingmei Biological Technology Co., Ltd., China) was used according to the manufacturer’s instructions.

#### Statistical method

The data were processed and analyzed by using SPSS 26.0 software. If the data followed the normal distribution, they were presented as mean ± SD (standard deviation). Data were compared for differences using two independent samples t-tests or one-way ANOVA. If the data were not normally distributed, they were presented as median (IQR, interquartile range), and a non-parametric test was adopted, and *p* < 0.05 was considered statistically significant.

## Results

### Clinical experiments

#### Participant’s characteristics

As illustrated in Table 1, the ages of the treatment group and control group were 57.30 ± 7.82 years and 56.94 ± 7.95 years, respectively. There was no significant difference in age between the two groups (*p* = 0.97). Among the treatment group, 4 males (13.33%) and 26 females (86.67%). While in the control group, 5 were males (16.13%) and 26 were females (83.87%). There was no significant difference in gender between the two groups (*p* = 0.76). The disease duration of the treatment and control groups were 46.33 ± 21.34 months and 45.29 ± 20.92 months, respectively. There was no significant difference in disease duration between the two groups (*p* = 0.84). In grading of severity of KOA assessed by Kellgren-Lawrence score grading, 6 cases were grade I, 20 were grade II, and 4 were grade III among the treatment group. While, in the control group, 7 cases were grade I, 19 were grade II, and 5 were grade III. There was no significant difference in severity of KOA between the two groups (*p* = 0.99). The joint function grades in the treatment group included 10 cases of grade I, 18 cases of grade II, and 2 cases of grade III. In comparison, the control group had 11 cases of grade I, 18 cases of grade II, and 2 cases of grade III. There was also no significant difference in joint function between the two groups (*p* = 0.87). Therefore, the age, gender, disease duration, severity of arthritis, and joint function of the two groups of patients are comparable, and follow-up research can be carried out.

**TABLE 1:** Comparison of baseline characteristics between treatment group and control group.

	Treatment group (n = 30)	Control group (n = 31)	*p*-value
Age, yrs, mean ± SD	57.30 ± 7.82	56.94 ± 7.95	0.97
Female sex, n (%)	26 (86.67)	26 (83.87)	0.76
Disease duration, months, mean ± SD	46.33 ± 21.34	45.29 ± 20.92	0.84
Grading of severity of KOA			
Ⅰ, n (%)	6 (20.00)	7 (22.58)	0.99
Ⅱ, n (%)	20 (66.67)	19 (61.29)	
Ⅲ, n (%)	4 (13.33)	5 (16.13)	
Grading of joint function			
Ⅰ, n (%)	10 (33.33)	11 (35.48)	0.87
Ⅱ, n (%)	18 (60.00)	18 (58.06)	
Ⅲ, n (%)	2 (0.07)	2 (6.45)	

Note: there were no significant differences in baseline characteristics between therapy group and control group.

#### Efficacy evaluation

**FIGURE 1:**
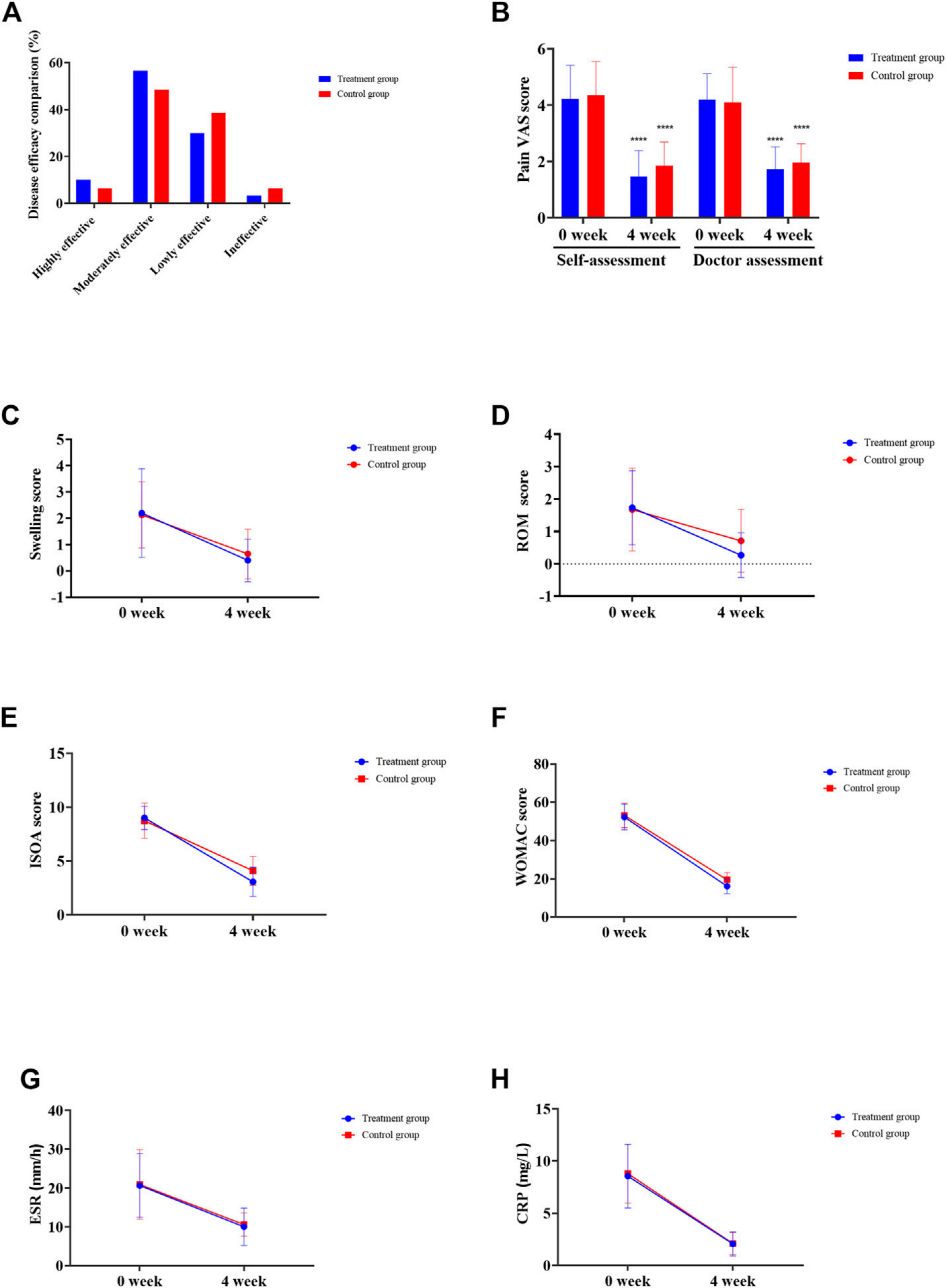
Clinical efficacy assessment. **(A)** Disease effectiveness comparison. **(B)** Joint pain VAS score. **(C)** Joint swelling score. **(D)** Joint range of motion (ROM) score. **(E)** Michel Lequesen index of severity for osteoarthritis (ISOA) score. **(F)** WOMAC score. **(G)** Erythrocyte sedimentation rate (ESR). **(H)** C-reactive protein (CRP). *p < 0.05, **p < 0.01, ***p < 0.001, ****p < 0.0001.

The clinical observation results showed that 3 (10%) cases were highly effective, 17 (56.67%) cases were moderately effective, 9 (30%) cases were lowly effective, and 1 (3.33%) case was ineffective, with a total effective rate of 96.67% in the treatment group. In the control group, 2 (6.45%) cases were highly effective, 15 (48.39%) cases were moderately effective, 12 (38.71%) cases were lowly effective, and 2 (6.45%) cases were ineffective, and the total effective rate was 93.55% (all *p* > 0.05) (Figure 1A). The VAS score of patients in the treatment group was 4.23 ± 1.19 before administration and 1.46 ± 0.93 after 4 weeks of administration (*p* = 2.81 × 10^−14^). The VAS score of patients in the control group was 4.35 ± 1.20 before administration and 1.86 ± 0.83 after 4 weeks of administration (*p* = 1.14 × 10^−13^). In the treatment group, the VAS score of physician was 4.20 ± 0.92 before administration and 1.73 ± 0.79 after 4 weeks of administration (Figure 1B, *p* = 5.44 × 10^−16^). In the control group, the VAS score of physician was 4.10 ± 1.25 before administration, and the VAS score of physician was 1.97 ± 0.66 after 4 weeks of administration (Figure 1B, *p* = 1.07 × 10^−11^). In the treatment group, the medium of swelling score was 2.00 before administration and 0.00 after 4 weeks of administration (Figure 1C, *p* = 1.80 × 10^−5^). The swelling score of the control group was 2.00 before administration and 0 after 4 weeks of administration (Figure 1C, *p* = 1.10 × 10^−5^). In the treatment group, the joint range of motion (ROM) was 2.00 before administration and 0.00 after 4 weeks of administration (Figure 1D, *p* = 9.40 × 10^−7^). The ROM in the control group was 2.00 before administration and 0.00 after 4 weeks of administration (Figure 1D, *p* = 0.002). In the treatment group, ISOA score was 9.00 ± 1.80 before administration and 3.07 ± 1.36 after 4 weeks of administration (Figure 1E, *p* = 8.49 × 10^−21^). The ISOA in the control group was 8.74 ± 1.63 before administration and 4.10 ± 1.33 after 4 weeks of administration (Figure 1E, *p* = 4.78 × 10^−18^). In the treatment group, the WOMAC score was 52.27 ± 6.74 before administration and 16.13 ± 3.85 after 4 weeks of administration (Figure 1F, *p* = 3.24 × 10^−33^). The WOMAC score of the control group was 53.13 ± 6.38 before administration and 19.52 ± 3.71 after 4 weeks of administration (Figure 1F, *p* = 9.10 × 10^−34^). In the treatment group, the ESR was 20.67 ± 8.21 (mm/h) before administration and 10.03 ± 4.80 (mm/h) after 4 weeks of administration (Figure 1G, *p* = 7.21 × 10^−18^). The ESR of the control group was 20.90 ± 8.94 (mm/h) before administration and 10.61 ± 3.07 (mm/h) after 4 weeks of administration (Figure 1G, *p* = 1.90 × 10^−7^). In the treatment group, the CRP was 8.56 ± 3.05 (mg/L) before administration and 2.06 ± 1.15 (mg/L) after 4 weeks of administration (Figure 1H, *p* = 3.12 × 10^−15^). The CRP of the control group was 8.80 ± 2.81 (mg/L) before administration and 2.09 ± 1.06 (mg/L) after 4 weeks of administration (Figure 1H, *p* = 2.71 × 10^−17^). After drug intervention, the joint function grades in the treatment group included 22 cases of grade I, 8 cases of grade II, and 0 cases of grade III, while the control group had 19 cases of grade I, 12 cases of grade II, and 0 cases of grade III and no significant difference was observed (*p* = 0.97).

#### Safety evaluation

Compared with before treatment, there was no significant difference in safety indicators such as blood cell analysis (white blood cell, red blood cell, hemoglobin, platelet), liver function (alanine transaminase, aspartate aminotransferase), renal function (blood urea nitrogen, creatinine), and electrocardiogram after 4 weeks of treatment (all *p* > 0.05) (Table 2). Our research has proved that YQYXF is safe for KOA patients.

**TABLE 2:** Safety index evaluation.

Project	Group	Before treatment	After 4 weeks of treatment	*p*-value
WBC (×10^9^/L)	Treatment group	6.03 ± 1.17	5.58 ± 1.04	0.12
	Control group	5.10 ± 0.63	5.14 ± 0.51	0.77
RBC (×10^9^/L)	Treatment group	4.65 ± 0.28	4.70 ± 0.43	0.59
	Control group	4.63 ± 0.52	4.84 ± 0.51	0.88
Hb (g/L)	Treatment group	141.17 ± 7.30	141.47 ± 8.51	0.88
	Control group	139.23 ± 10.70	143.94 ± 7.75	0.12
PLT (×10^9^/L)	Treatment group	225.00 ± 41.55	217.40 ± 57.96	0.56
	Control group	216.06 ± 43.89	228.45 ± 54.87	0.34
ALT (U/L)	Treatment group	18.80 ± 7.83	16.87 ± 6.97	0.32
	Control group	16.71 ± 7.92	19.26 ± 6.40	0.17
AST (U/L)	Treatment group	21.57 ± 5.34	19.17 ± 5.36	0.09
	Control group	18.71 ± 5.81	19.97 ± 4.18	0.33
BUN (mmol/L)	Treatment group	4.80 ± 1.46	4.72 ± 1.22	0.82
	Control group	5.00 ± 1.54	4.47 ± 1.02	0.12
Cr (umol/L)	Treatment group	67.20 ± 11.74	69.10 ± 14.08	0.57
	Control group	67.00 ± 12.84	67.58 ± 11.10	0.85

WBC, white blood cell; RBC, red blood cell; Hb, hemoglobin; PLT, platelet; ALT, alanine transaminase; AST, aspartate aminotransferase; BUN, blood urea nitrogen; Cr, creatinine, all *p* > 0.05.

### Animal experiment

#### Screening active components of YQYXF with UHPLC-QE-MS

**FIGURE 2:**
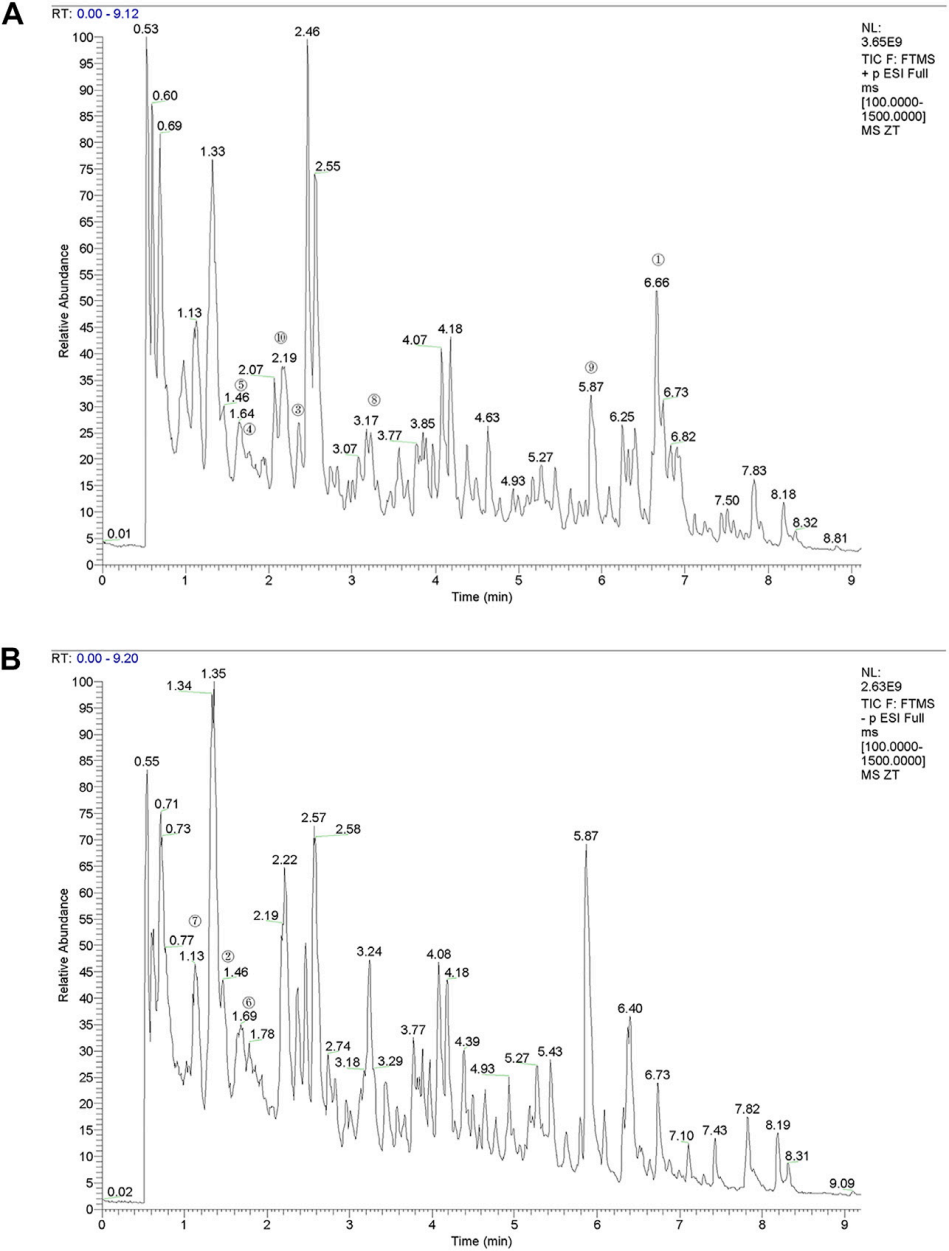
The total ion chromatograms (TIC) of YQYXF that were obtained in positive ion mode and negative ion mode. **(A)** UHPLC-QE-MS analysis base peak intensity chromatograms of YQYXF in positive ion mode. **(B)** UHPLC-QE-MS analysis base peak intensity chromatograms of YQYXF in negative ion mode.

The compounds in YQYXF were identified by the UHPLC-QE-MS method. A total of 447 compounds were characterized in YQYXF (Figure 2A: positive ion modes; Figure 2B, negative ion modes). Among these, 17 kinds of components were detected, including flavonoids, phenols, terpenoids, amino acid derivatives, phenylpropanoids, alkaloids, aromaticity aliphatic acyl, xanthones, jasmonic acid, organic acids and derivatives, fatty acids, prenol lipids, lipoic acids and derivatives, carboxylic acids and derivatives, carbohydrates and derivatives, alkaloids, and quinones. The peak area represents the relative abundance of the substance, and ppm is the deviation between the measured mz value of the substance and the theoretical mz value. ppm = (measured mz value - theoretical mz value) × 1,000,000 ÷ theoretical mz value. The leading substances were defined as the top 10 substances identified by UHPLC-QE-MS analysis. As in Table 3, the top ten substances were ranked from highest to lowest according to their respective peak area. The larger the peak area, the higher the ranking.

**TABLE 3:** Identification of components of YQYXF by UHPLC-QE-MS analysis (top 10).

Name	Composite score	Rtmed (s)	Mzmed	ppm	Formula	Peak area
3,5,7,8-tetramethoxy-2-(3,4,5-trimethoxyphenyl)chromen-4-one	0.78	399.66	433.15	1.04	C_22_H_24_O_9_	3.17×10^9^
2″-O-beta-L-galactopyranosylorientin	0.63	88.34	609.15	2.69	C_27_H_30_O_16_	1.66×10^9^
Herbacetin	1.00	132.90	303.05	1.25	C_15_H_10_O_7_	1.40×10^9^
Isoliquiritigenin	0.99	101.62	257.08	3.29	C_15_H_12_O_4_	1.32×10^9^
Biochanin-7-O-glucoside	0.90	98.83	447.13	0.00	C_22_H_22_O_10_	1.25×10^9^
Liquiritin	0.72	102.92	417.12	3.15	C_21_H_22_O_9_	1.06×10^9^
4-Methoxysalicylic acid	0.93	63.18	167.04	0.40	C_8_H_8_O_4_	9.81×10^8^
Formononetin-7-O-glucoside	0.87	190.81	431.13	0.96	C_22_H_22_O_9_	9.71×10^8^
Licoricesaponin H2	0.90	352.46	823.41	2.05	C_42_H_62_O_16_	8.85×10^8^
Naringin	0.61	130.20	581.18	4.13	C_27_H_32_O_14_	8.51×10^8^

#### Effects of YQYXF in KOA rats

**FIGURE 3:**
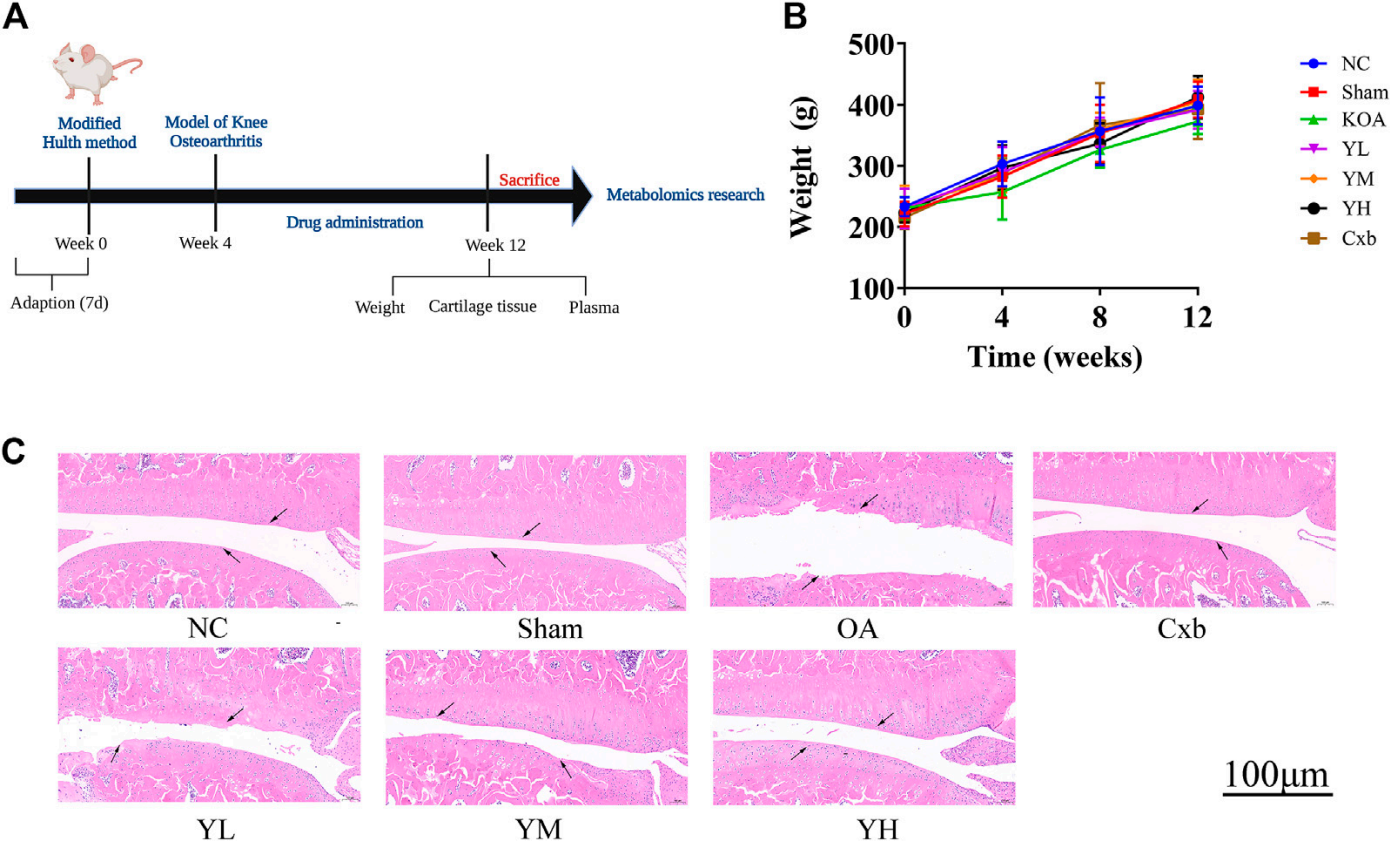
Effects of YQYXF in OA rats. **(A)** Time flow chart. **(B)** Effects of YQYXF on body weight. **(C)** Cartilage tissue HE staining. NC: normal control group, sham: sham operation group, OA: model group, Cxb: celecoxib, YL: low-dose groups of YQYXF, YM: medium-dose groups of YQYXF, YH: high-dose groups of YQYXF.

The time flow chart of the experiment is shown in Figure 3A. The body weight of the rats in each group showed a steadily increasing trend (Figure 3B). The overall growth trend of the body weight of the KOA group was lower than that of the NC group. The weight gain trend of Cxb and YQYXF groups with different doses was higher than that of KOA group, but there was no significant difference in the body weight (*p* > 0.05). The point indicated by the arrow was the cartilage surface (Figure 3C). The NC and sham operation groups’ cartilage surfaces were smooth and flat, without cracks and defects. Chondrocytes were orderly arranged, clearly stratified, and evenly distributed, without obvious cell clusters. In the model group, the cartilage surface ulcer became thinner, and the local cartilage calcification layer was ruptured and disappeared. Chondrocytes were disordered. The stratification was not easy to recognize, and regional cell clusters were apparent. The Cxb group’s cartilage surface was smooth, without obvious cracks and defects, and the chondrocytes were arranged in order with a regular hierarchical structure. The cartilage surface was rough in the YQYXF low-dose (YL) group, and some cartilage tissues were defective. The cartilage surface was not smooth in the YQYXF medium (YM) dose group. Some cartilages were defective or cracked, and the chondrocytes were complex and disordered. The hierarchical structure was obvious, and local cell clusters appeared. The staining matrix was uniform. The cartilage surface in the YQYXF high-dose (YH) group was smooth, without apparent defects and tear.

#### Effects of YQYXF on potential biomarkers in KOA rats

**FIGURE 4:**
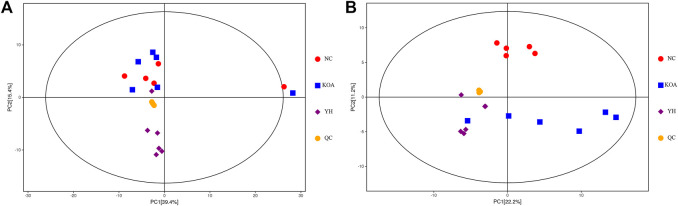
PCA score plots of the QC, NC, KOA, and YH groups. **(A)** Positive ion modes. **(B)** Negative ion modes. NC group (n = 5), KOA group (n = 6), YH group (n = 6).

To further explore the effect of YQYXF on the endogenous differential metabolites and their related metabolic pathways of KOA, we used the plasma of rats in the NC group, KOA group, and YH group for metabolomic analysis. We included QC samples throughout the experimental process to ensure the stability and reliability of the data and the system. The QC samples (yellow dots) were closely clustered together, indicating that the UHPLC-QE-MS system had good stability and was reliable for metabolomic analysis of the samples. LC-MS data obtained from the plasma samples were analyzed using PCA for metabolic changes between the NC, KOA, YH, and QC samples (Figure 4A: positive ion modes; Figure 4B, negative ion modes). In both the positive and negative ion modes, we found a large deviation in 1 rat in the NC group, and no meaningful results could be drawn. Therefore, we excluded it from further analysis. The contribution ratio of principal component 1 (PC1) was 39.4%, and that of PC2 was 15.4% (Figure 4A: positive ion modes). The contribution ratio of principal component 1 (PC1) was 22.2%, and that of PC2 was 11.2% (Figure 4B, negative ion modes). In the positive ion mode, the separation of KOA and NC samples was insignificant. It is worth noting that the KOA samples were significantly separated from the NC samples in the negative mode plot, indicating significant metabolic differences between the two groups. Meanwhile, YH and OA samples were separated, but the separations were not significant in both positive and negative ion modes. Due to the complex multidimensional characteristics of metabolic data, unsupervised PCA model analysis alone could not well distinguish group differences among samples. The OPLS-DA model was employed to characterize the differential metabolites among NC, KOA, and YH groups to further identify the differences in the composition of the metabolites. *R* ^*2*^ indicated how well the variation of a variable was explained, and *Q* ^*2*^ meant how well a variable could be predicted. The replacement test established the corresponding OPLS-DA model to obtain the *R* ^*2*^ and *Q* ^*2*^ values of the random model by randomly changing the order of the classification variable Y and repeating it for several times (n = 200), which played an essential role in avoiding the over-fitting of the test model and evaluating the robustness of the model.

**FIGURE 5:**
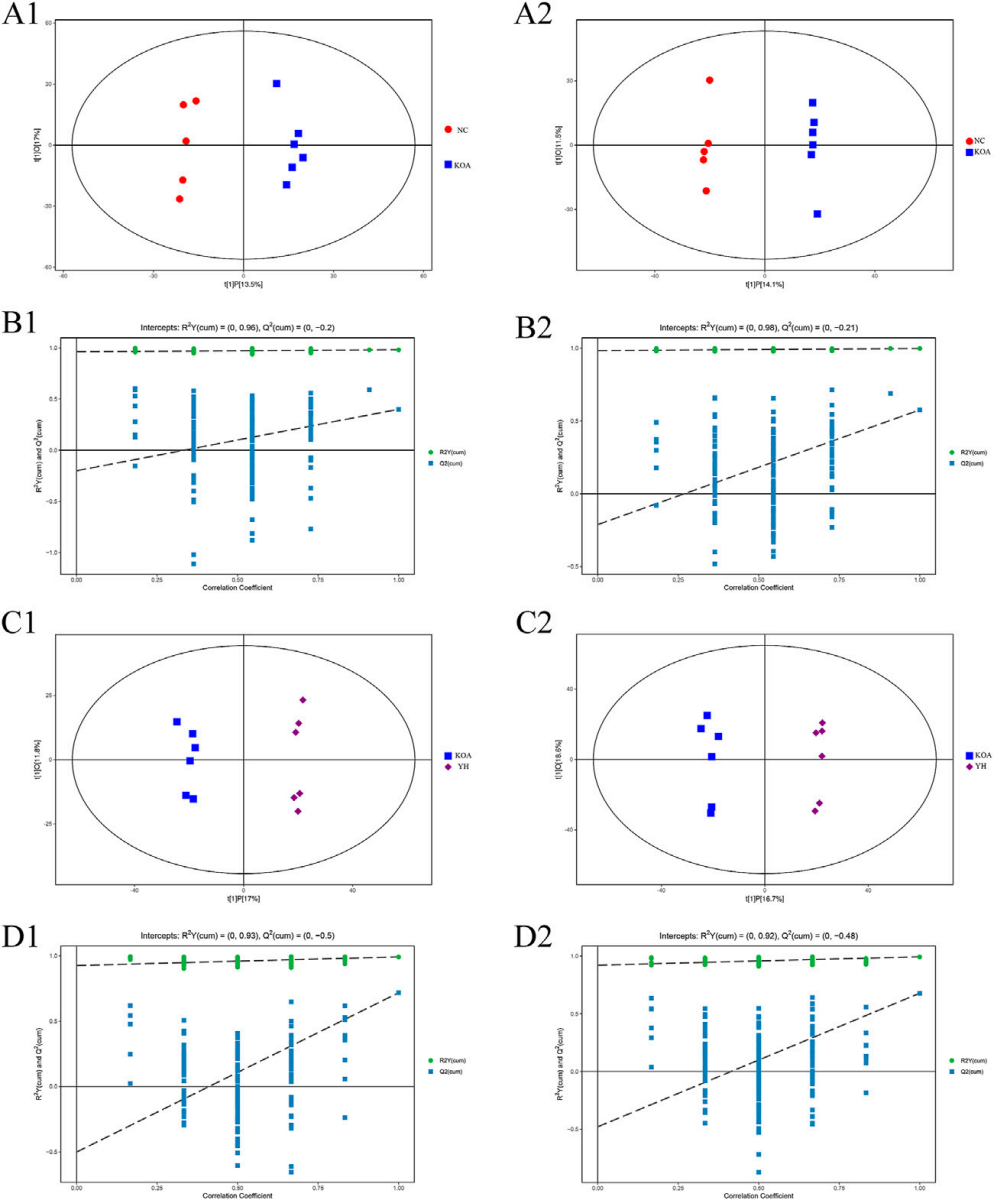
OPLS-DA analysis of serum of mice. OPLS-DA scores plots: KOA vs*.* NC (A1: positive ion, A2: negative ion), YH vs*.* KOA (C1: positive ion, C2: negative ion). Permutation test of OPLS-DA model: KOA vs*.* NC (B1: positive ion, B2: negative ion); YH vs*.* KOA (D1: positive ion, D2: negative ion).

The OPLS-DA score plots showed an obvious separation between the KOA and NC group in the positive and negative ion mode from the metabolic profiles (Figure 5, A1: positive ion, A2: negative ion). The permutation test results *R* ^*2*^ *Y* and *Q* ^*2*^ between the KOA and NC groups were respectively 0.98 and 0.40 in the positive ion mode and 0.99 and 0.58 in negative ion mode (Figure 5, B1: positive ion, B2: negative ion). The YH and KOA groups OPLS-DA scores plots were also well separated in the positive and negative ion mode (Figure 5, C1: positive ion, C2: negative ion); *R* ^*2*^ *Y* and *Q* ^*2*^ were respectively, 0.99 and 0.72 in the positive ion mode and 0.99 and 0.68 in the negative ion mode (Figure 5, D1: positive ion, D2: negative ion). All *R* ^*2*^ *Y* were very close to 1, indicating that the established model conformed to the real situation of the sample data. The intercept between the regression line of *Q* ^*2*^ and the longitudinal axis was less than zero. Meanwhile, with the gradual reduction of displacement retention, the proportion of the Y variable of displacement increased, and the *Q* ^*2*^ of random model gradually decreased. It showed that the model in this study had good robustness, and there was no over-fitting phenomenon. Therefore, the OPLS-DA results further confirmed the successful establishment of our OA rat model and that YH administration could regulate the metabolic profile of the rat.

**FIGURE 6:**
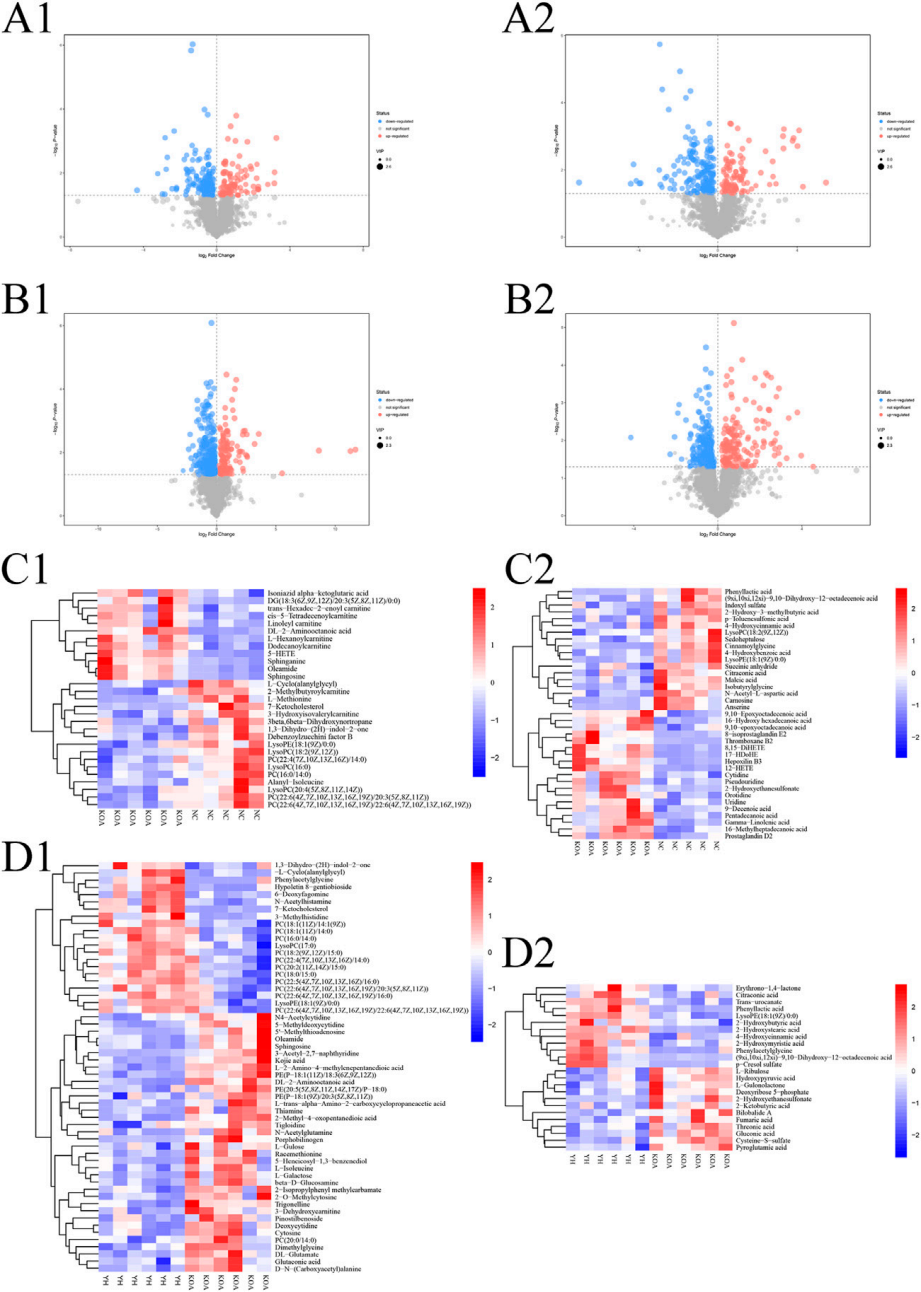
Multivariate statistical analysis of metabolite profiles in plasma. Volcano plot of comparison groups: KOA vs*.* NC (A1: positive ion, A2: negative ion); YH vs*.* KOA (C1: positive ion, C2: negative ion). Heatmap of comparison groups: KOA vs*.* NC (C1: positive ion, C2: negative ion); YH vs*.* KOA (D1: positive ion, D2: negative ion). Screening of differential metabolites by metabolomic analysis. Significantly upregulated metabolites are shown in red, significantly downregulated metabolites in blue, and non-significantly different metabolites in grey.

To investigate the contribution of potential biomarkers between the two groups, a volcano plot was drawn, followed by Student’s t-test. Volcano plot of comparison groups: KOA vs*.* NC (Figure 6, A1: positive ion; A2: negative ion); YH vs*.* KOA (Figure 6, B1: positive ion; B2: negative ion). It summarized the contribution of each variable to the model. The metabolites with VIP>1 and *p* < 0.05 were considered as significantly changed metabolites. In total, 66 metabolites were identified in the heatmap of hierarchical clustering analysis between the KOA and NC groups, including 29 and 37 metabolites in the positive and negative ion modes, respectively. Between the YH and KOA groups, 81 metabolites were identified, including 57 and 24 metabolites in the positive and negative ion modes, respectively. Heatmap of comparison groups: KOA vs*.* NC (Figure 6, C1: positive ion, C2: negative ion); YH vs*.* KOA (Figure 6, D1: positive ion, D2: negative ion). Taking the intersection of differential metabolites between the KOA vs*.* NC group and the YH vs*.* KOA group, there were 16 potential biomarkers, including 8 lipids and lipid-like molecules, 2 organic acids and derivatives, 2 phenylpropanoids and polyketides, 2 oganoheterocyclic compounds, 2 oganic acids and derivatives (Table 4).

**TABLE 4:** Identified Potential Biomarkers Regulated by Yiqi Yangxue formula (YQYXF).

Name	MS2 score	Rt (s)	Mz	VIP (KOA vs. NC)	*p*-value (KOA vs. NC)	Fold change (KOA vs. NC)	VIP (YH vs. KOA)	*p*-value (YH vs. KOA	Fold change (YH vs. KOA)	AUC (KOA vs. NC)	AUC (YH vs. KOA)	CV (%)	Ion mode	KOA vs. NC	YH vs. KOA
1,3-Dihydro-(2H)-indol-2-one	0.99	28.61	134.06	1.61	0.002	0.32	1.30	0.03	2.16	0.83	0.86	63.53	+	**↓**	↑
Oleamide	0.98	82.95	282.28	2.38	0.01	3.60	1.72	0.02	0.50	1.00	0.92	71.07	+	↑	**↓**
Sphingosine	0.97	82.95	300.29	2.40	0.01	3.71	1.71	0.02	0.48	1.00	0.92	73.62	+	↑	**↓**
DL-2-Aminooctanoic acid	0.96	243.66	160.13	2.39	0.001	3.20	2.17	0.01	0.32	1.00	1.00	66.90	+	↑	**↓**
LysoPE (18:1 (9Z)/0:0)	0.93	220.69	480.31	1.74	0.03	0.67	1.53	0.03	1.41	0.75	0.92	29.06	+	**↓**	↑
7-Ketocholesterol	0.90	31.98	401.34	2.44	0.03	0.19	2.23	0.01	4.62	1.00	1.00	88.44	+	**↓**	↑
PC(22:6 (4Z,7Z,10Z,13Z,16Z,19Z)/20:3 (5Z,8Z,11Z))	0.78	152.05	856.58	1.91	0.01	0.65	1.52	0.04	1.33	0.89	0.83	49.78	+	**↓**	↑
PC(22:4 (7Z,10Z,13Z,16Z)/14:0)	0.68	159.91	782.57	1.74	0.04	0.76	1.4	0.04	1.23	0.92	0.86	58.83	+	**↓**	↑
L-Cyclo (alanylglycyl)	0.62	379.50	129.07	2.02	0.004	0.50	1.76	0.01	2.03	0.97	0.89	60.36	+	**↓**	↑
PC(16:0/14:0)	0.61	169.58	706.54	1.86	0.02	0.64	1.59	0.02	1.37	0.89	0.89	27.85	+	**↓**	↑
PC(22:6 (4Z,7Z,10Z,13Z,16Z,19Z)/22:6 (4Z,7Z,10Z,13Z,16Z,19Z))	0.56	148.69	878.57	2.07	0.01	0.41	1.63	0.04	1.62	0.94	0.81	48.93	+	**↓**	↑
2-Hydroxyethanesulfonate	0.98	160.46	124.99	1.82	0.05	2.29	1.74	0.04	0.40	0.81	0.92	64.01	-	↑	**↓**
Phenyllactic acid	0.97	125.42	165.05	2.40	0.03	0.13	2.27	0.003	3.57	0.86	1.00	95.37	-	**↓**	↑
Citraconic acid	0.96	453.31	129.02	1.79	0.03	0.70	1.54	0.04	1.41	0.81	0.83	28.08	-	**↓**	↑
(9xi,10xi,12xi)-9,10-Dihydroxy-12-octadecenoic acid	0.93	178.74	313.24	1.87	0.02	0.39	1.87	0.04	7.68	0.78	0.94	125.36	-	**↓**	↑
4-Hydroxycinnamic acid	0.68	58.39	163.04	2.00	0.01	0.66	1.87	0.005	1.41	0.89	0.92	22.82	-	**↓**	↑

**FIGURE 7:**
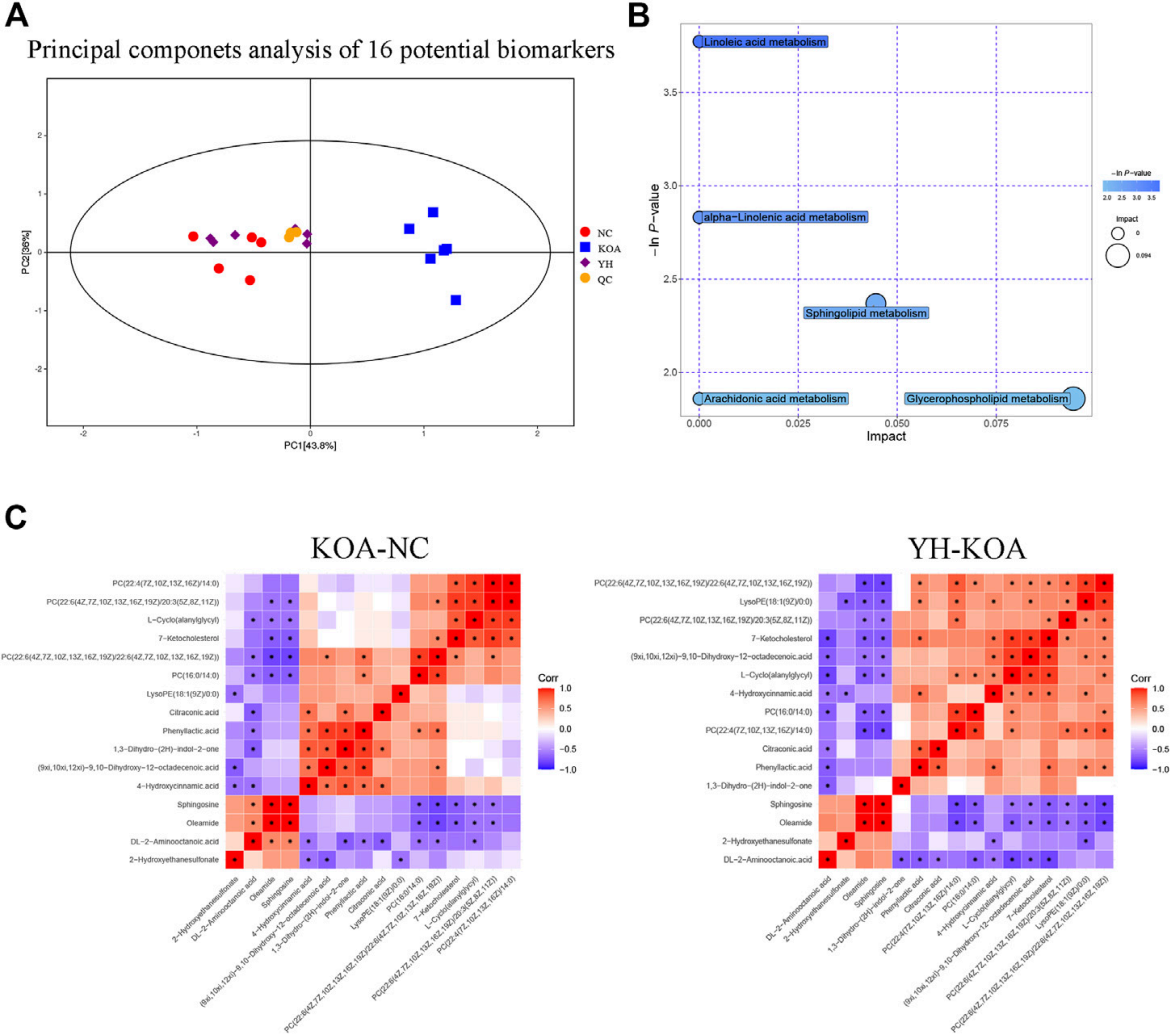
Metabolic profiling of 16 potential biomarkers. **(A)** Principal components analysis score plot of 16 differential metabolites. **(B)** Metabolic pathway bubble plot of 16 differential metabolites. **(C)** Heatmap of correlation analysis. The horizontal and vertical coordinates in the figure represent the contrasting differential metabolites. Red represents a positive correlation, blue represents a negative correlation, and the darker the color, the stronger the correlation. Significant correlations are marked with an asterisk (*).

In order to more intuitively express the intervention effect of YQYXF components on the screened metabolites, PCA analysis was performed on the 16 screened potential biomarkers (Figure 7A). The intra-group aggregation and inter-group dispersion were obvious in the NC and the KOA group, indicating that the plasma metabolites in the KOA rats were distinguished from the NC. The KOA group was significantly separated from the NC group and YH group, while the NC group and YH group were significantly aggregated, indicating that YH can regulate KOA-related metabolites. Further, we mapped the differential metabolites to authoritative metabolite databases. After obtaining the matching information of the differential metabolites, we searched and analyzed the metabolic pathway of the pathway database of *Rattus norvegicus* (rat). The metabolic pathway enrichment analysis of differential metabolites showed that YQYXF mainly exerted its anti-inflammatory effect by regulating five pathways: linoleic acid metabolism, α-linolenic acid metabolism, sphingolipid metabolism, arachidonic acid metabolism, and glycerophospholipid metabolism (Figure 7B). In addition, we performed correlation analysis on 16 biomarkers. Internal interaction and crosstalk networks are established between phenylpropanoic acids and organoheterocyclic compounds, fatty acyls and organic sulfonic acids, carboxylic acids and diazinanes, fatty acyls and organonitrogen compounds (Figure 7C).

#### YQYXF modulates oxidative stress and lipid peroxidation capacity in KOA rats

**FIGURE 8:**
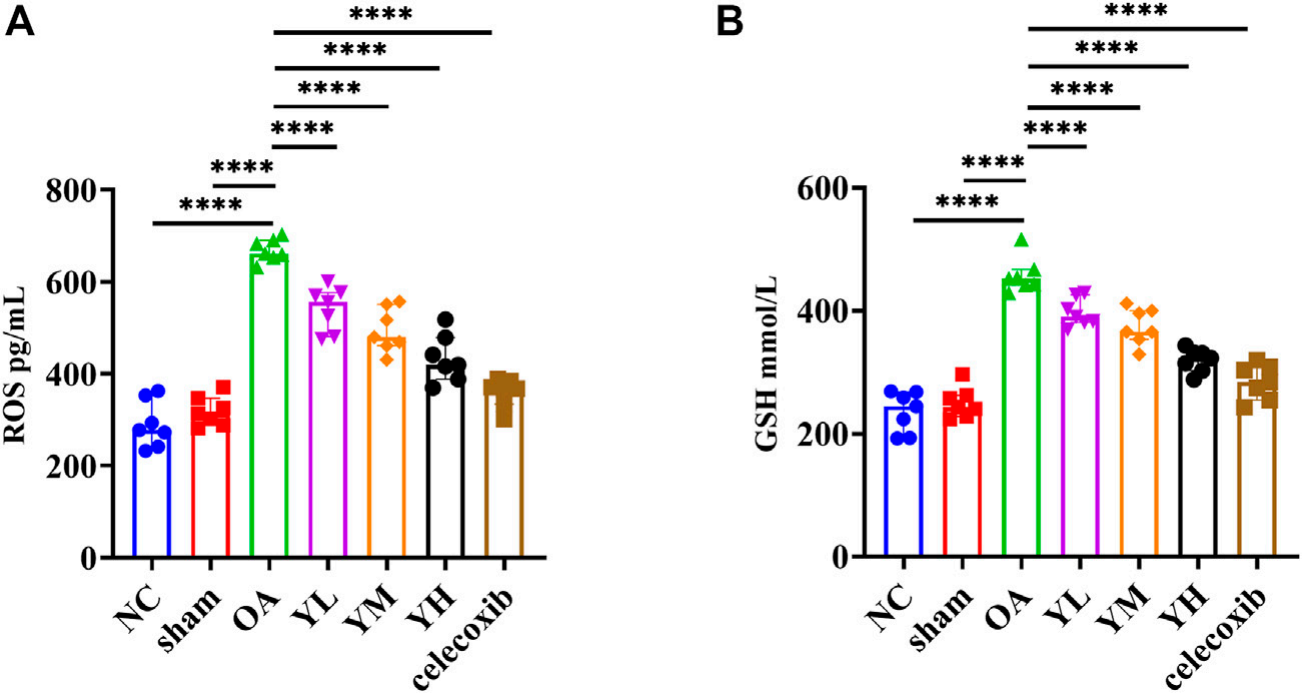
The effect of YQYXF on anti-oxidative stress and lipid peroxidation in OA rats. **(A)** Levels of ROS in plasma. **(B)** Levels of GSH in plasma. All data were expressed as mean ± SD. *p < 0.05, **p < 0.01, ***p < 0.001, ****p < 0.0001.

The results of metabonomics experiments showed that YQYXF mainly regulated the signal pathway related to lipid metabolism. Therefore, it may play the role of anti-KOA cartilage destruction by repairing lipid metabolism disorder. We further explored the changes of oxidative stress and lipid peroxidation-related factors in KOA rats. The GSH of NC, sham, OA, YL, YM, YH, and Cxb group was 235.69 ± 33.23, 250.43 ± 24.85, 467.97 ± 29.25, 397.37 ± 22.89, 374.49 ± 29.29, 319.31 ± 18.88, 284.02 ± 28.56 mmol/L, respectively. The ROS of NC, sham, OA, YL, YM, YH, and Cxb group was 290.41 ± 50.77, 318.59 ± 31.99, 681.62 ± 25.38, 540.76 ± 48.31, 495.07 ± 47.84, 433.13 ± 51.34, 359.10 ± 30.95 pg/mL, respectively. Compared with the NC and sham group, the expression of GSH (*p* = 1.59 × 10^−19^ for the KOA group vs*.* the NC group, *p* = 1.67 × 10^−18^ for the KOA group vs*.* the sham group) and ROS (*p* = 9.23 × 10^−21^ for the KOA vs*.* the NC group, *p* = 1.43 × 10^−19^ for the KOA group vs*.* the sham group) were significantly increased (Figures 8A, B). The GSH of drug-treated groups was lower than that of the KOA group (p = 1.60 × 10^−5^ for the low-YQYXF-dose group, p = 8.6826 × 10^−8^ for the middle-YQYXF-dose group, p = 5.00 × 10^−13^ for the high-YQYXF-dose group, and p = 5.50 × 10^−16^ for the Cxb group) and the ROS of drug-treated groups was lower than that of the KOA group (p = 1.76 × 10^−7^ for the low-YQYXF-dose group, p = 2.34 × 10^−10^ for the middle-YQYXF-dose group, p = 5.74 × 10^−14^ for the high-YQYXF-dose group, and p = 9.69 × 10^−18^ for the Cxb group), suggesting that the YQYXF could regulate the plasma lipid metabolism disorder in the KOA rats. Hence, YQYXF may exert anti-KOA cartilage degeneration by regulating lipid peroxidation.

## Discuss

Clinical research results showed that YQYXF could improve the clinical symptoms of KOA patients without noticeable adverse reactions. In animal experiments, the 66 potential disease biomarkers of KOA and 81 related metabolites of YQYXF were screened out. After comparative analysis, there were 16 potential biomarkers in the intervention of YQYXF in KOA rats, and exert anti-KOA through five metabolic pathways, including sphingolipid metabolism, glycerophospholipid metabolism, linoleic acid metabolism, α-linolenic acid metabolism, and arachidonic acid metabolism.

Sphingolipids display important functions in various pathologies such as obesity, diabetes, and OA (El Jamal et al., 2020). Sphingosine 1-phosphate (S1P) is a metabolite of cell membrane sphingolipids enriched in circulating fluid. It binds to G protein-coupled S1P receptors to regulate embryonic development and organ function. S1P binding triggers multiple cellular and physiological events, including the localization of immune cells to sites of inflammation and regulation of T-cell differentiation (Th17 and Treg cells) (Robinson et al., 2022). The balance between the levels of S1P and sphingosine has been considered as a switch that could determine whether a cell proliferates or dies (El Jamal et al., 2020). Masuko found that S1P may play a unique role in the pathophysiology of KOA by regulating VEGF expression in chondrocytes (Masuko et al., 2012). A study found that the activity of sphingosine kinase 1 increased with osteoclast differentiation, and its expression was enhanced in the subchondral bone of mice with KOA (Cherifi et al., 2021). The lipid mediator S1P was identified as a therapeutic target for KOA (Stradner et al., 2013; Ustyol et al., 2017). In this study, potential biomarkers were significantly enriched in the sphingolipid metabolism pathway, suggesting that YQYXF may reduce KOA cartilage damage and improve lipid metabolism mainly by regulating sphingolipid metabolism changes.

Glycerophospholipids form the essential lipid bilayer of all biological membranes and are intimately involved in signal transduction, regulation of membrane trafficking, and many other membrane-related phenomena (Farooqui et al., 2000). The alterations in phospholipid composition and concentrations are associated with the development of KOA (Kosinska et al., 2014; Zhang W. et al., 2014). A study found activation of glycerophospholipid metabolism and oxidative stress pathways in synovial fluid metabolism in patients with KOA (Carlson et al., 2019). In this study, YQYXF may improve lipid metabolism by affecting the level of glycerophospholipids, alleviating the progression of KOA.

In addition, linoleic acid, α-linolenic acid, and arachidonic acid all belong to polyunsaturated fatty acids (PUFAs). While all PUFAs reduced markers of oxidative stress, omega-3 PUFAs additionally decreased prostaglandin production (Loef et al., 2019). The omega-3 PUFAs have been shown to decrease markers of inflammation and cartilage degradation. Oxidative stress can be directly assessed by measuring ROS. Known ROS include superoxide, hydrogen peroxide, peroxyl radicals, and reactive nitrogen species (including nitric oxide and peroxynitrite derived from the nitric oxide) (Duan L. et al., 2020). Previous studies have found that excessive ROS generated by lipid metabolism disorders can induce chondrocyte apoptosis (Poulet and Staines, 2016). Excessive accumulation of ROS can cause chondrocyte damage and cartilage matrix degradation, promoting the occurrence of KOA (Bolduc et al., 2019). ROS can also destroy proteoglycans and type II collagen in the cartilage matrix by activating matrix metalloproteinases, inhibiting matrix synthesis, and leading to loss of cartilage integrity (Mehana et al., 2019). GSH can exert a destructive effect on ROS through an enzymatic mechanism, reducing the level of ROS or inhibiting its activity. Imbalanced ROS/GSH may result from a direct increase of ROS, consumption of GSH, intracellular oxidoreductase interference, or thioredoxin activity reduction (Liu et al., 2022). GSH and ROS in the model group were significantly increased, suggesting an imbalance between ROS production and elimination. Furthermore, the intervention of different doses of YQYXF may activate the feedback regulation mechanism, promote the reduction of ROS level, and then lead to the corresponding decrease of GSH, which can alleviate the imbalance. Thus, YQYXF may reduce ROS production by balancing lipid metabolism disorders and inhibiting KOA cartilage destruction. However, further experimental verification is still needed.

A study used liquid chromatography/mass spectrometry (LC/MS)-based metabolomics to explore the serum metabolomics in rats with KOA. The six biomarkers were identified, which were metabolized through tryptophan metabolism, glutamate metabolism, nitrogen metabolism, spermidine metabolism, and fatty acid metabolism pathways (Zhao et al., 2021). Another study used ultra-high performance liquid chromatography-triple quadrupole mass spectrometry (UPLC-TQ-MS), followed by multivariate statistical analysis, to determine the serum amino acid profiles of KOA patients and healthy controls. The metabolic pathways with the most significant effects were involved in the metabolism of alanine, aspartate, glutamate, arginine, and proline (Chen et al., 2018). In our study, the plasma metabolites of KOA and control group participated in alanine, aspartate and glucose metabolism, pyrimidine metabolism, and biosynthesis of unsaturated fatty acids. The results are consistent with the findings of the above studies. In addition, the fasting serum of KOA patients and healthy controls was assessed by metabolomic analysis (Tootsi et al., 2020). The changes in the serum levels of amino acids, sphingomyelins, phoshatidylcholines and lysophosphatidylcholines of the KOA patients compared with healthy controls suggest systemic inflammation in severe KOA patients. In our study, YQYXF is mainly involved in lipid metabolism pathways, such as sphingolipids metabolism and glycerol phospholipids metabolism, which indicates that YQYXF may play a role in treating systemic inflammation in KOA.

For note, the flavocoxid is a medical food consisting of plant-derived flavonoids which have anti-inflammatory activity and are used to treat chronic KOA. Studies have shown that flavocoxid was as effective as naproxen in managing the signs and symptoms of KOA (Levy et al., 2010). In our study, 99 flavonoids in YQYXF were identified, including naringin, baicalin, icariin, and quercetin. Naringin, a natural flavanone found in citrus fruits, and its aglycone have been demonstrated to ameliorate obesity and dyslipidemia. The principal mechanisms by which these flavonoids exert their action involve upregulation of peroxisome proliferator activated receptor α and adenosine 5′-monophosphate-activated protein kinase, and the downregulation of genes involved in lipid metabolism (Massaro et al., 2022). Naringin is an effective therapeutic drug for the treatment of KOA and KOA-related symptoms, which can support the recovery of hind-limb weight-bearing (Xu et al., 2017). Naringin can prevent cartilage destruction in KOA by inhibiting the nuclear factor kappa-B (NF-κB) signaling pathway, which reduces Tumor necrosis factor-α (TNF-α)-mediated chondrocyte inflammation and cartilage matrix degradation (Zhao et al., 2016). Baicalein can ameliorate inflammatory-related apoptotic and catabolic phenotypes in human chondrocytes (Zhang X. et al., 2014). In addition, the anti-inflammatory and anti-apoptotic effects of baicalein are mediated by inhibiting the translocation of phosphorylated p65 to the nucleus (Li et al., 2017). Icariin has been shown to stimulate osteogenic differentiation and bone formation and to increase the synthesis of the cartilage extracellular matrix (Zhao et al., 2019). A study has demonstrated that the IKBKB, NFKBIA, MAPK8, MAPK9, and MAPK10 may be the hub genes affected by icariin when providing its beneficial effects on KOA. In addition, icariin can alleviate KOA by inhibiting NOD-like receptor thermal protein domain associated protein 3-mediated pyroptosis (Zu et al., 2019). Quercetin may be related to the inhibition of interleukin-1β (IL-1β) and TNF-α production *via* the Toll-like receptor 4/NF-κB pathway in KOA rats (Zhang et al., 2020). The use of quercetin partially abrogated intestinal flora disorder and reversed fecal metabolite abnormalities (Lan et al., 2021). In addition, 32 phenolic compounds were found in YQYXF, such as paeonol, 4-methylatechol, and thymol. Paeonol, as an essential component in traditional Chinese medicine, has anti-inflammatory activity and can offer therapy for a multitude of inflammatory-related diseases. Studies have shown that applying paeonol can attenuate the secretion of cartilage extracellular matrix and cartilage degrading enzymes induced by IL-1β in chondrocytes (Liu et al., 2017). Besides, paeonol can also alleviate destabilization of the medial meniscus-induced articular cartilage degeneration *in vivo* (Liu et al., 2017). There are few studies on phenolic compounds in OA, which need further research and verification.

For note, the metabolic pathways likely contribute to symptoms and pathology in KOA. Some studies demonstrated that metabolites in the synovial fluid and blood could be used as biomarkers for KOA incidence, prognosis, and response to therapy (Rockel and Kapoor, 2018). Various metabolites can directly influence the perception of pain. Secreted phospholipase A2 catalyzes the conversion of phosphatidylcholine (PC) analogues to lysoPC analogues. Subsequent metabolism of lysoPCs *via* autotaxin generates lysophosphatidic acid, an inflammatory and pain-producing signal. Endocannabinoids, endogenously produced lipid-derived compounds, could be beneficial for individuals with KOA to reduce pain symptoms. In turn, the endocannabinoids may result in the production of lysoPCs, which could promote joint pain due to metabolism of lysophosphatidic acid (Muccioli, 2010). In addition, the oxidized linoleic acid metabolite and partial TRPV1 agonist 9-hydroxyoctadecandienoic acid was shown to be involved in chronic inflammatory pain (Wedel et al., 2022). The linoleic acid can attenuate inflammatory responses and reduce LPS-induced phosphorylation of proteins associated with NF-κB signaling (Kim et al., 2020). In addition, the dysregulation of sphingolipid metabolism contributes to neuropathic pain (Stockstill et al., 2018) (Table 5).

**TABLE 5:** The relevance of metabolic pathways to symptoms and pathology.

Pathway	Symptoms and pathology
Linoleic acid metabolism	Regulate inflammatory reactions and pain, and maintain the stability of blood glucose and blood fat levels
Alpha-Linolenic acid metabolism	Regulate lipid metabolism and inflammatory reactions
Sphingolipid metabolism	Regulate inflammatory reactions and pain
Arachidonic acid metabolism	Participate in immune and inflammatory reactions
Glycerophospholipid metabolism	Regulate lipid metabolism

The limitation of this study is that the components of traditional Chinese medicine are complex, with the characteristics of multiple targets and pathways. Although the components of YQYXF were identified, the analysis of members entering the blood and the study of pharmacokinetics still need to be clarified.

In conclusion, YQYXF could be an effective and promising agent for treating KOA, which might exert its action by regulating multiple lipid metabolism-related pathways. Our study provides new insights into studying the underlying molecular mechanism of YQYXF against oxidative stress in the KOA model. However, further research exploration is needed.

## Data Availability

The original contributions presented in the study are publicly available. This data can be found here: https://www.ebi.ac.uk/metabolights/. Accession number: MTBLS5667.
